# Bimetallic Biogenic Pt-Ag Nanoparticle and Their Application for Electrochemical Dopamine Sensor

**DOI:** 10.3390/bios13050531

**Published:** 2023-05-10

**Authors:** Muhammed Bekmezci, Hudanur Ozturk, Merve Akin, Ramazan Bayat, Fatih Sen, Rozhin Darabi, Hassan Karimi-Maleh

**Affiliations:** 1 Sen Research Group, Department of Biochemistry, Faculty of Art and Science, Dumlupinar University, Kutahya 43100, Turkey; 2 Department of Materials Science & Engineering, Faculty of Engineering, Dumlupinar University, Evliya Celebi Campus, Kutahya 43100, Turkey; 3 School of Resources and Environment, University of Electronic Science and Technology of China, Xiyuan Ave, Chengdu 611731, China

**Keywords:** antibacterial activity, Pt-Ag bimetallic nanoparticle, *Curcuma longa*, dopamine sensor

## Abstract

In this study, Silver-Platinum (Pt-Ag) bimetallic nanoparticles were synthesized by the biogenic reduction method using plant extracts. This reduction method offers a highly innovative model for obtaining nanostructures using fewer chemicals. According to this method, a structure with an ideal size of 2.31 nm was obtained according to the Transmission Electron Microscopy (TEM) result. The Pt-Ag bimetallic nanoparticles were characterized using Fourier Transform Infrared Spectroscopy (FTIR), X-ray Diffractometry (XRD), and Ultraviolet-Visible (UV-VIS) spectroscopy. For the electrochemical activity of the obtained nanoparticles in the dopamine sensor, electrochemical measurements were made with the Cyclic Voltammetry (CV) and Differential Pulse Voltammetry (DPV) methods. According to the results of the CV measurements taken, the limit of detection (LOD) was 0.03 µM and the limit of quantification (LOQ) was 0.11 µM. To investigate the antibacterial properties of the obtained Pt-Ag NPs, their antibacterial effects on *Escherichia coli* (*E. coli*) and *Staphylococcus aureus* (*S. aureus*) bacteria were investigated. In this study, it was observed that Pt-Ag NPs, which were successfully synthesized by biogenic synthesis using plant extract, exhibited high electrocatalytic performance and good antibacterial properties in the determination of dopamine (DA).

## 1. Introduction

Nanotechnology makes our lives easier by changing and controlling the size and structure of materials at the nanoscale [1,2,3,4,5], makes our lives easier by being involved with the production, design, characterization, and implementation of various devices and systems [6,7,8,9,10]. Nanomaterials are materials with a range of 1–100 nm [7,11,12]. Parts with at least one external dimension at the nanoscale are defined as nanoparticles, and materials composed of these nanoparticles are called nanomaterials [13,14]. Nanomaterials are used in many areas such as health, energy, defense, and sensors due to their many properties [15,16,17,18,19,20]. For example, nanomaterials are used in drug delivery systems, magnetic fluid hyperthermia, nanoparticle-based imaging, and tissue regeneration. In addition, studies with nanomaterials allow for developments in the field of biosensors and the design of sensors in the health field [14,21]. As a result of advances in the field of nanomaterials [22], studies involving increasing the sensitivity of the sensor, keeping it stable for a long time, and obtaining inexpensive structures have been carried out [23,24,25]. With the development of nanomaterials, the daily use of biosensors has also increased. Today, there are sensors developed against biomolecules such as glucose, ascorbic acid, and dopamine. Thanks to the sensors developed, biomolecules can be detected, and early diagnosis and treatment of diseases can be provided.

DA, an important biomolecule of our body, is a neurochemical transporter in our brain, located in the catecholamine and phenethylamine families, and controls our remembering, learning, attitudes, mood, and focus [26]. Dopamine, which acts on the renal, cardiovascular, endocrine, and central nervous systems, should be present in the blood and serum at a rate of 10^−6^–10^−9^ mol/L. Disproportionate levels of dopamine have been found in diseases such as Parkinson’s, schizophrenia, depression, and addiction [27]. At this point, accurate and fast detection of DA is important and electrochemical sensors are frequently used for DA detection. As a result of literature research, it has been seen that noble metal nanoparticles such as palladium (Pd), silver (Ag), gold (Au), and platinum (Pt) are used in electrochemical sensors designed to measure DA levels. The high surface area of metal nanoparticles and their high electrocatalytic activity has enabled them to be used successfully in the field of sensors [28].

Within the scope of the study, the Pt nanoparticle was used as the main metal, due to the superior electrocatalytic activity and conductivity of Pt metal, in order to develop a sensor against the DA biomolecule. However, due to the high cost of Pt metal, to reduce the cost and improve its properties, Ag metal was added, and a Pt-Ag bimetallic structure was synthesized and used. The reason why Ag was preferred as the second metal in our study is that it shows biocompatibility with Pt, is easily available, is low cost, and has sustainable electrocatalytic activity properties. Ag is also frequently used for antibacterial applications, as it is effective against viruses, fungi, and bacteria, and has antibacterial properties [25]. Silver ions (Ag^+^) released by Ag nanoparticles prevent the replication of microbial DNA, thus suppressing the expression of ribosomal proteins and enzymes involved in ATP hydrolysis [26]. In addition, Ag ions can adhere to the cytoplasmic membrane and cell walls and increase the permeability of the membrane and disrupt the bacterial envelope [29]. Thus, they kill microbes. Ag nanoparticles are also used in many fields such as biomedical imaging, biosensing, and drug delivery.

Various physical and chemical methods such as electrochemical reduction, chemical reduction, heat evaporation, and photochemical reduction have been developed for the production and stabilization of metal nanoparticles [30]. However, these methods cause the formation of harmful by-products, in addition to being expensive [31]. The green synthesis method uses natural resources such as plant materials, bacteria, fungi, and algae that do not harm the environment [32,33]. It is a more economical and more environmentally friendly alternative method to physical or chemical methods that harm the environment. It is known that ideal size structures can be obtained with this method. Therefore, in this study, Pt-Ag bimetallic nanoparticles were synthesized by the green synthesis method using *C. longa*, and the characterization of the obtained material was investigated using FTIR, XRD, and UV-Vis. The biogenic reduction method enabled the formation of the nanostructure without the need for other reductants, high temperatures, or advanced processes. Briefly, electrochemical sensors were applied to Pt-Ag bimetallic nanoparticles produced by the green synthesis method for DA determination. Although the basis of the study involved it being used as an electrocatalyst, the obtained biogenically derived structure was found to be an ideal antibacterial. Following this purpose, the antibacterial properties of gram-negative (*E. coli*) and gram-positive (*S.aureus*) bacteria were investigated.

## 2. Materials and Methods

### 2.1. Chemicals

Silver Nitrate (AgNO_3_), Platinum (II) Chloride (PtCl_2_), DA and Phosphate-buffered saline (PBS), and methanol (CH_3_OH) J.T. Baker were obtained from Sigma Aldrich (Burlington, Massachusetts, MA, USA), and Methanol (CH_3_OH) obtained from J.T. Baker (Pittsburgh, Pennsylvania, PA, USA) with 99.9% purity. A PBS solution (pH 3), distilled water (dH_2_O), and alcohol were used in this study. All of the glass materials used in the study were cleaned with dH_2_O, and the alcohol was dried before use. *C. longa* used in the study was purchased commercially from the local market, and the dried powder form of the plant was used.

### 2.2. Synthesis of Pt-Ag Nanoparticle

10 g of powdered *C. longa* plant was taken, and dH_2_O was added and the mixture was boiled in the microwave. The solution was then filtered with filter paper to obtain the extract. dH_2_O was added to the resulting extract at a ratio of 4:1. Next, 32 mg of AgNO_3_ and 32 mg of PtCl_2_ (10 mM), and AgNO_3_ (10 mM) were added to the extract. The resulting solution was stirred at 100 °C and 395 rpm until it changed from light orange to black. A color change was observed after 36 h. Next, washing with dH_2_O was initiated. The washed solution was then kept in an oven at 45 °C for 24 h for drying, and bimetallic powder NP was obtained.

### 2.3. Characterization of Pt-Ag Nanoparticles

FTIR (Perkin Elmer-Operational Qualification, Shelton, Connecticut, CT, USA), XRD (Rigaku Miniflex, Texas, TX, USA), UV-VIS (Perkin Elmer Lamda750, Shelton, Connecticut, CT, USA), and TEM (JEOL-Jem 2100, Akishima, Tokyo, Japan) were used for the characterization of Pt-Ag bimetallic nanoparticles. Characterization studies were performed by determining the functional groups and elemental transitions of Pt-Ag NPs.

### 2.4. Electrochemical Characterization

Electrochemical methods are very promising in studies with sensors [34,35,36]. Electrical changes occur due to the reduction/oxidation reactions of the analyte, and different methods are used to analyze these electrical changes [37]. In this study, electrochemical characterizations of Pt-Ag NPs obtained from the *C. longa* plant were investigated by cyclic voltammetry (CV), differential pulse voltammetry (DPV), and scan rate (SR) methods using a potentiostat galvanostat device (Gamry reference 3000, Warminster, Pennsylvania, PA, USA).

### 2.5. Antibacterial Characterization

Solutions of 3 different concentrations, 20, 50, and 100 concentrations, were formed from Pt-Ag bimetallic NPs obtained with the *C. longa* plant extract. These solutions were applied to gram-negative bacteria *E. coli* and gram-positive bacteria *S. aureus* bacteria in a sterile cabinet. The absorption measurements of the inhibition percentages they showed were performed with the Multimode Reader (TriStar2 S LB 942) device, and their antibacterial activities were examined.

## 3. Conclusion and Discussion

### Ultraviolet-Visible (UV-VIS) Spectroscopy, FTIR, and XRD Characterization

**Figure 1:**
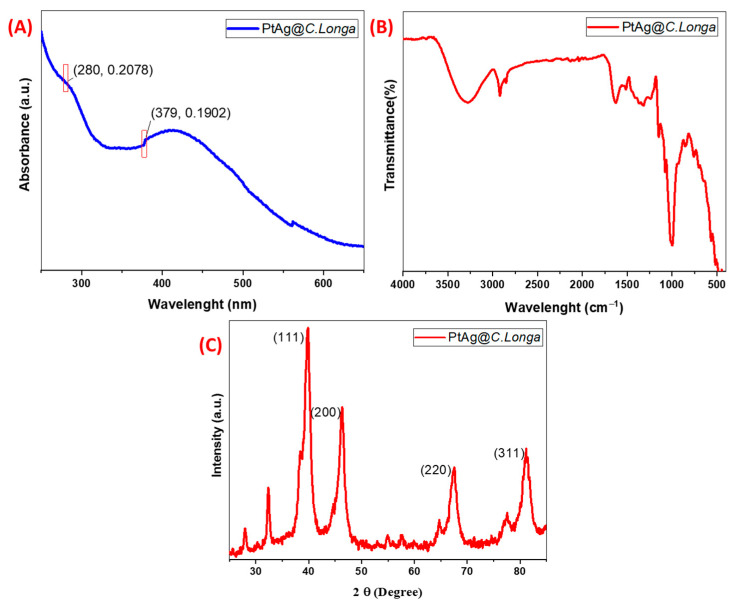
(**A**) UV-VIS spectrum plot of biogenically synthesized Pt-Ag bimetallic NPs; (**B**) FTIR representation of Pt-Ag bimetallic NPs; (**C**) XRD plot of Pt-Ag bimetallic NPs.

The study continued with material characterization. The first of these is UV-VIS. UV-vis, which is frequently used in the characterization of nanoparticles, allows us to confirm the formation of nanoparticles by measuring the surface plasmon resonance. These are σ–σ * (star transition from sigma to sigma), n–σ * (star transition from n to sigma), π–π * (star transition from pi to pi), n–π * (from n to pi) star transit) can be grouped [38]. In this study, UV-Vis measurement was made and the obtained graphic was interpreted according to electronic transitions. The UV-VIS spectrum graph is shown in Figure 1A. It was observed that the Ag nanoparticle gave a maximum absorption signal at 379 nm in the UV spectrum of the Pt-Ag bimetallic nanoparticle, and a shift was observed in the Ag peak due to the biogenic synthesis method of Ag NPs [39]. Another strong surface plasmon resonance was observed at 280 nm, which is the characteristic wavelength range of Pt NPs.

Another characterization method used in the study is FTIR analysis. FTIR is frequently used for surface characterization by showing the characteristic functional groups of nanoparticles from spectral bands, and is based on the interaction of electromagnetic radiation with molecules. The analysis (FTIR spectrum) performed for this purpose is shown in Figure 1B. FTIR is frequently used for the surface characterization of nanoparticles by showing their characteristic functional groups from spectral bands. It is based on the principle of the interaction of electromagnetic radiation with molecules. The FTIR spectrum of Pt-Ag bimetal synthesized from the *C. longa* plant is shown in Figure 1B. When the Ag nanoparticle was characterized by FTIR, it was observed that it showed O-H stretching between 3441 cm^−1^ [40]. In the Pt spectrum, a sharp peak was observed in the 1728 cm^−1^ region due to C=O stretching, in the 1365 cm^−1^ region due to C-H stretching, and in the 1219 cm^−1^ region due to C-N stretching [41]. Due to the presence of -OH groups at 3340 cm^−1^, the Pt-Ag nanoparticle has C-H bonds at 2921 cm^−1^, at 1715 cm^−1^ due to C=O double bonds, at 1370 cm^−1^ due to O-H bonds, and at 1030 cm^−1^. It was observed that it gave a strong peak which originated from the C-C bonds at cm^−1^ [42].

XRD is a powerful method frequently used in research. The XRD method is preferred in the study of crystal variants and structures, phase analysis, and the particle size of nanomaterials. This method is based on the diffraction of X-rays that occur in materials, especially crystalline materials. In this study, characterization was performed with the XRD method. In Figure 1C, the graph of the data obtained by XRD analysis of Pt-Ag NPs synthesized with the *C. longa* extract is given. As seen in the graph, the highest peak for Pt-Ag was observed in the region of 2θ = 39.2°. This region corresponds to the (111) crystal plane of the face-centered cubic phase [43]. Other diffraction peaks occur at the 45.9°, 67.1°, and 80.2° regions corresponding to the (200), (220), and (311) planes [44]. According to the result of the XRD graph, the particle size was also calculated according to the Debye Scherer equation. According to the Debye Scherrer formula, approximately D = 4.08 nm was obtained. The results were close to the particle size obtained in the TEM.

**Figure 2:**
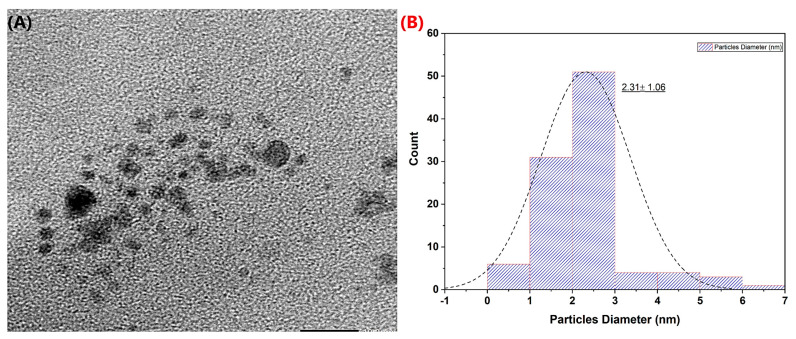
TEM image and particle distribution histogram of biogenically synthesized Pt-Ag Bimetallic NPs. ((**A**) TEM image, (**B**) Histogram graphic).

To support the results obtained and to examine the morphological distribution of the Pt-Ag material, it was characterized by TEM (Figure 2). It was observed that the synthesized Pt-Ag material had a spherical morphology. As seen in the histogram plot of the particle size distribution, its approximate size was 2.31 nm. According to the TEM image, it was noticed that there may be dimensional differences caused by different particles in the bimetallic NP formation mechanism, which is related to the formation mechanism. In particular, it appeared that Ag NP was in a larger form in the particle distribution, and changed the Standard deviation.

## 4. Electrochemical Characterization

### 4.1. Use of Pt-Ag Bimetallic Modified Electrode

**Figure 3:**
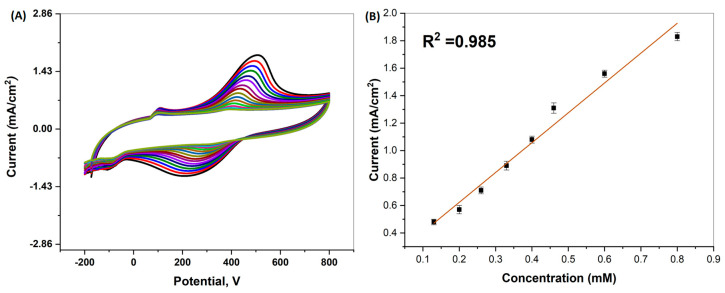
(**A**) CV concentration plot of biogenically synthesized Pt-Ag Bimetallic NPs; (**B**) Concentration plot of biogenically synthesized Pt-Ag Bimetallic NPs calculated according to CV analysis.

CV is an electrochemical method and one of the best-known and most effective methods for modification characterization [45,46]. It was used for the determination of the electrochemical characterization in this study. Glase carbon electrode (GCE) was diluted with 10 µL of DMF, dH_2_O, and nafion with the help of a micropipette on the tip surface, and Pt-Ag bimetallic NPs were dropped. Our working electrode was then left in an oven at 45 °C for 30 min to dry. A PBS solution was added to the cell, and the dried GCE, reference electrode, and counter electrode were placed in the cell, and measurements were taken. CV measurements were taken in the 0.1–0.8 mM concentration range, as shown in Figure 3A. As seen in Figure 3, it was observed that the peaks obtained as a result of the experiment increased in direct proportion to the amount of dopamine added. In the presence of Pt-Ag bimetallic NP, it was observed that dopamine was reduced in the range of 0–400 V, and oxidation peaked in the range of 400–600 V. These results showed that the obtained sensor was effective at detecting dopamine. As seen in Figure 3B, it was observed that the Pt-Ag CV electrochemical oxidation currents increased linearly according to the concentration range of 0.1–0.8 mM. The R^2^ value was calculated as 0.978.

### 4.2. Effect of Scan Rate

**Figure 4:**
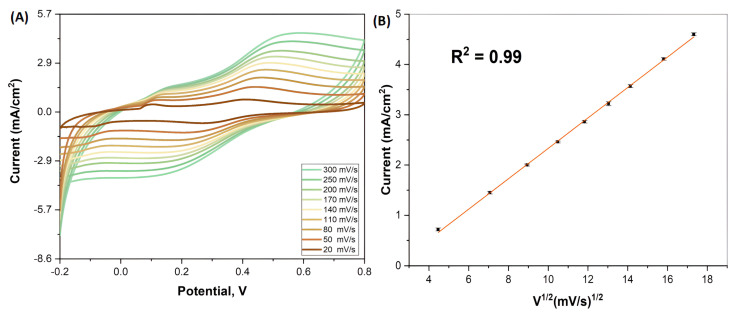
(**A**) SR graph of biogenically synthesized Pt-Ag Bimetallic Nps, (**B**) Concentration graph of biogenically synthesized Pt-Ag Bimetallic Nps calculated according to SR analysis.

The scan rate graph of Pt-Ag bimetallic NPs is shown in Figure 4. As can be seen in Figure 4, the study was carried out in the scanrate range of 20–300 mV/s. In the obtained results, it was observed that the oxidation peak intensity increased as the scan rate increased. In short, it was observed that the current increased as the rate increased. In addition, these current anodic peaks obtained were calculated according to the square root of time and exhibited a good linear graph. This result showed that the obtained sensor is compatible with the diffusion-controlled model.

### 4.3. Studying Differential Pulse Voltammetry (DPV)

**Figure 5:**
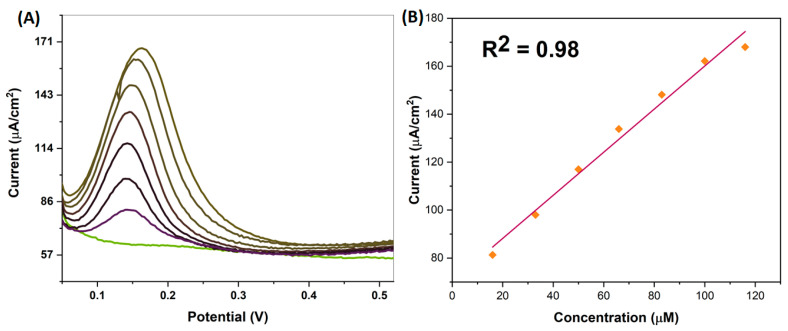
(**A**) DPV plot of biogenically synthesized Pt-Ag NP; (**B**) Concentration plot of biogenically synthesized Pt-Ag NP calculated according to DPV analysis.

DPV is a precision measurement method, and it was used to determine the sensitive electrochemical oxidation of Pt-Ag NPs with DPV in this study. A PBS buffer solution (pH 3.0) was used in our experiment. Before starting the experiment, the electrodes were cleaned and 10 µL of Pt-Ag bimetallic NPs diluted with DMF, dH_2_O, and nafion were dropped onto the working electrode. It was then left to dry in an oven and measurements were taken. Measurements were determined to be in the range of 0–0.6 mV and a concentration range of 16 μM–0.11 mM. The Differential Pulse Voltammetry (DPV) plot and the calculated concentration plot are shown in Figure 5. Looking at the results, it was observed that as the dopamine concentration increased, an increase was observed in the anodic peak flow points (Figure 5A). The results supported the CV experiments, and the increase in currents was more clearly observed with the DPV study. In the study, a rightward shift was observed in the peak currents, which is thought to be related to the increase in concentration. The resulting current peak points were plotted according to the concentration, and the R^2^ calculation was made (Figure 5B). According to the data obtained, the LOD and LOQ values calculated in the 16 μM–0.11 mM concentration range were found to be 0.03 µM and 0.11 µM, respectively.

In the last stage of the study, the results were compared with other studies in the literature. These literature surveys and our results are summarized in Table 1. This comparison showed that the fabricated biogenic Pt-Ag electrocatalyst exhibited a favorable electrochemical performance over a wide range of DA concentrations with a detection limit very close to that found in the literature. This is since Pt-Ag NPs obtained by biogenic methods are as good as those obtained by other synthesis methods.

## 5. Bacterial Inhibition Rate of Pt-Ag Nps

The *E. coli* and *S. aureus* bacteria used in our study were first placed into the medium in the tube. Afterward, the tubes were left in the shaker for 24 h at 37 °C for the bacteria to multiply. The gram-negative and gram-positive control groups were placed in 96-well plates. The propagated *E. coli* and *S. aureus* bacteria were used at three different concentrations: 100 μg/mL, 50 μg/mL, and 25 μg/mL. Pt-Ag NPs were added to the bacteria to test their inhibition. To examine the bacterial inhibition at 100 μg/mL, 50 μg/mL, and 25 μg/mL concentrations, specific concentrations of the NP and medium were added. The plate on which the additions were made was left at 37.5 °C for 20 h, and bacteria were grown. In our experiment, the inhibition range of Pt-Ag bimetallic NPs synthesized by *C. longa* against bacteria was examined.

**Figure 6:**
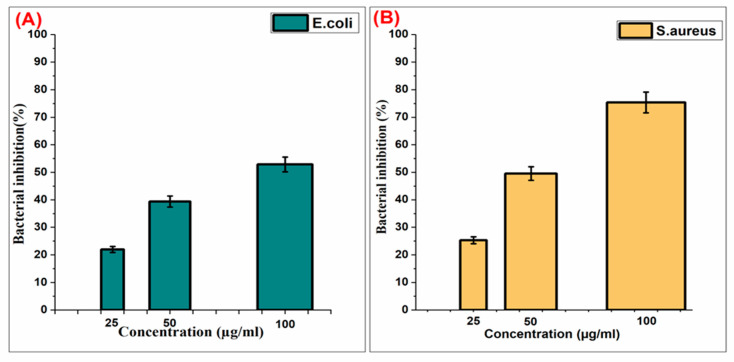
(**A**) Pt-Ag NPs *E. coli* bacterial inhibition plot; (**B**) Pt-Ag NPs *S. aureus* bacterial inhibition plot.

The inhibition graph showing the antibacterial properties of the Pt-Ag bimetallic NPs we synthesized because of the experiment are shown in Figure 6.

In the study, it was observed that the inhibition rate in *E. coli* bacteria, a gram-negative bacterium, was 58% in the presence of Pt-Ag NPs at 100 μg/mL concentration, 43% in the presence of NP at 50 μg/mL concentration, and 23% in the presence of NP at 25 μg/mL concentration. The inhibition rate observed in the presence of NP at 100 μg/mL concentration in *S.aureus* bacteria, which is a Gram-positive bacterium, was 78%; it was 53% in the presence of NP at a concentration of 50 µg/mL, and it was observed that it was 27% in the presence of NP at a concentration of 25 μg/mL. Pt-Ag alloys induce a more controlled release of Ag+ ions than pure Ag shells, so they can remain as antibacterial agents for a longer period of time. Our data were found to be similar to other studies [53]. As a result of the experiment, it was seen that there was a direct correlation between the NP concentration and the percentage of bacteria inhibition, which showed that Pt-Ag NPs could be used for antibacterial studies.

## 6. Conclusions

In this study, Pt-Ag bimetallic nanoparticles were successfully synthesized by the biogenic reduction method using the *C. longa* plant extract. Thanks to this method, ideal-sized nanostructures were obtained without the need for heavy chemicals and advanced processes. The synthesized Pt-Ag bimetallic nanoparticles were characterized using FTIR, TEM, XRD, and UV-VIS spectroscopy. The most important finding in the characterization was that the ideal nanostructure for biogenic reduction was obtained at 2.31 nm. The main aim of the study was to use the catalyst in electrolyzes. However, as in a biogenic reduction, the antibacterial applications of the catalyst were also investigated, taking economic concerns into account. According to the results obtained by CV, DPV, and SR in electrochemical analysis methods, the NPs showed high electrocatalytic performance for dopamine determination. The LOD was found to be 0.03 µM and the LOQ was 0.1 µM. In the antibacterial analysis, Pt-Ag bimetallic NPs showed good antibacterial properties on *E. coli* and *S. aureus bacteria*. It was observed that the use of Pt-Ag led to more controlled Ag^+^ ion release and remained as an antibacterial agent for a longer time compared to pure Ag NPs. In this study, *C. longa* nanoparticles were obtained by the green synthesis method, were successfully applied to the DA sensor, and showed antibacterial activity.

## Figures and Tables

**Table 1:** Comparison of the analytical performance of the DA sensor, in which Pt-Ag NP obtained by biogenic synthesis is used as an electrocatalyst with other DA sensors.

Material	Detection Method	Linear Range	LOD	References
**AgNPs**	LSPR	10 nM–1 µM	0.058 µM	[47]
**Pt–Ag/Gr**	DPV	0.1–60 µM	0.012 μM	[48]
**Ni/Ag/Zn**	DPV	1–25 μM	0.3 μM	[49]
**PtAg/WS2/GCE**	Amperometry	0.6–1000 μM	0.2 μM	[50]
**Cit-AuNP@PDA@PGE**	DPV	0.5–7.0 μM	0.96 µM	[51]
**Ag/CuO/ITO**	CV	0.04–10 μM	7 nM	[52]
**Biogenic Pt-Ag**	DPV	16 μM–0.11 mM	0.03 µM	This work

## Data Availability

Data will be provided by the author.
